# qPCR analysis of bivalve larvae feeding preferences when grazing on mixed microalgal diets

**DOI:** 10.1371/journal.pone.0180730

**Published:** 2017-06-29

**Authors:** Kai Liao, Wenbi Chen, Runtao Zhang, Haibo Zhou, Jilin Xu, Chengxu Zhou, Xiaojun Yan

**Affiliations:** 1 Key Laboratory of Applied Marine Biotechnology, Ningbo University, Chinese Ministry of Education, Ningbo, Zhejiang, P. R. China; 2 Collaborative Innovation Center for Zhejiang Marine High-efficiency and Healthy Aquaculture, Ningbo University, Ningbo, Zhejiang, P. R. China; University of Helsinki, FINLAND

## Abstract

Characterization of the feeding preferences of bivalve larvae would help improving the bivalve aquaculture and hatchery by providing appropriate microalgal diets. However, inaccurate and laborious identification and counting of microalgal species have challenged the selective feeding of bivalves. In the present study, we developed a highly specific and sensitive assay using quantitative polymerase chain reaction (qPCR) to assess the selective feeding of bivalve larvae based on species-specific primers targeting to microalgal 18S rDNA sequences. The assay exhibited good specificity. The detection limits of the qPCR assay were 769, 71, 781 and 21 18S rDNA copies for *Chaetoceros calcitrans*, *Isochrysis galbana*, *Platymonas helgolandica* and *Nannochloropsis oculata*, respectively. Using such assay, we found that *C*. *calcitrans* and *I*. *galbana* were preferentially ingested, whereas *N*. *oculata* was preferentially rejected in biodeposits of four bivalve species, *Tegillarca gransa*, *Cyclina sinensis*, *Scapharca subcrenata* and *Sinonovacula constricta*. Furthermore, our growth experiments revealed that *C*. *calcitrans* and *I*. *galbana* could significantly promote the shell growth, whereas feeding of *N*. *oculata* resulted in poorer growth of four bivalve species. These data indicated that qPCR might be useful in screening of efficient and reliable microalgal species for each bivalve species, leading to improved bivalve aquaculture and hatchery.

## Introduction

Live microalgae are the fundamental food sources for bivalves [1–3]. Previous study has revealed that some bivalves can distinguish their food from various types of particles, preferentially ingesting the high-quality ones, while rejecting those undesirable such as pseudofeces [4]. Therefore, it is important that the microalgae chosen as foods for bivalve aquaculture are preferentially ingested, and the characterization of feeding preferences of bivalves should help improving the bivalve aquaculture and hatchery by providing appropriate microalgal diets [5].

Several technologies have been developed to assess the feeding selection of bivalves, including particle counter, electronic particle counter and flow cytometry [4]. However, the first two methods are highly laborious and time-intensive, and do not accurately differentiate microalgae cells with same particle size and shape [6–10]. The third approach has been widely applied to bivalves using chlorophyll pigments as tracers of microalgae. However, because chlorophyll pigment is class-specific, not species-specific, the technique is less effective when microalgal preys belong to the same class [11–12]. In addition, although isotope labeling has been used to provide information on the feeding preferences of bivalves [13], this technique requires time-consuming and expensive isotope labeling to distinguish microalgae species. More recently, quantitative polymerase chain reaction (qPCR) offers a rapid and quantitative analysis for the DNA of prey organisms, such as microalgae, and this technique has been successfully applied to qualitative studies of the selective feeding of copepods and insects [11,14]. Because DNA is much more prey-specific than microalgal pigments, microalgal DNA should have a much larger potential as a quantitative prey tracer. Furthermore, this genetic sequence-based approach is more precise and rapid compared with particle counter and isotope labeling due to the high sensitivity and simplicity of qPCR.

Ark shell (*Tegillarca gransa* and *Scapharca subcrenata*), Venus clam (*Cyclina sinensis*) and Chinese razor clam (*Sinonovacula constricta*) are four economically important bivalve species in China. The total aquaculture production of these four bivalve species in 2014 was 1,140,216 tons [15]. *C*. *sinensis* has differential filtration rate in response to different microalgal species [16]. In addition, Shen et al. [17] showed that absorption efficiency in *S*. *subcrenata* highly depends on the species of microalgae consumed. Until now, little information is available on the selective feeding of these bivalve species since all diets are provided as monoalgal cultures in the experiments[4, 16–20].

In the present study, we developed a highly specific and sensitive qPCR assay to assess the selective feeding of bivalve larvae based on species-specific primers targeting to microalgal 18S rDNA sequences. Using this assay, we determined the selective feeding of above-mentioned four bivalve species in the presence of mixed microalgae containing *Chaetoceros calcitrans*, *Isochrysis galbana*, *Platymonas helgolandica* and *Nannochloropsis oculata*. Then, we also examined the effects of four microalgal species on the growth performance of four experimental bivalve species in order to evaluate the application of the qPCR assay for microalgal diet screening of bivalves.

## Materials and methods

### Culture of microalgae

Microalgal species, including *C*. *calcitrans*, *I*. *galbana*, *P*. *helgolandica* and *N*. *oculata*, were selected from the Microalgal Culture Laboratory at Ningbo University. Culture medium consisted of filtered (0.45 μm) and autoclaved seawater (salinity 25−26‰) enriched with NMB3 medium (KNO_3_ 100 mg/L, KH_2_PO_4_ 10 mg/L, Fe-citrate·5H_2_O 3 mg/L, VB_1_ 6 μg/L, VB_12_ 0.05 μg/L). Sodium metasilicate (20 mg/L) was added as a silica source for culture of the diatom *C*. *calcitrans*. The microalgae were grown in 2,500-mL flasks at 20 ± 2°C under continuous illumination provided by cool white fluorescent tubes at an intensity of 160−180 μmol photons/m^2^/s for about 1 week. In exponential phase, the microalgae were transferred into a 10-L photobioreactor, and air with 2% CO_2_ was supplied to support the massive growth. The microalgae in exponential growth phase were harvested and used to feed larval bivalves. Each microalga was cultured in triplicate.

### Culture of bivalves and sample collection

#### Culture of bivalve larvae

**Fig 1:**
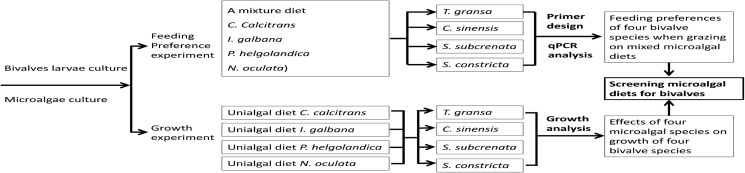
Schematic of experimental design.

Hatchery-reared larvae of *T*. *gransa*, *C*. *sinensis*, *S*. *subcrenata* and *S*. *constricta* were obtained from Yuejinyang Hatchery at Ninhai City, Zhejiang, China. They were cultured in a pool (length 8.0 × width 4.2 × depth 0.9 m) with continuous aeration after settlement. The seawater (salinity 20‰) was filtered by 1-m thick sand (diameter < 1 mm). Fresh sea-mud dried at 200°C and filtered by nylon cribrose silk (75 μm) was spread onto the pool bottom at a thickness of about 1−2 mm. Larvae were fed mixed microalgae (*C*. *calcitrans*, *I*. *galbana*, *P*. *viridis*, *P*. *helgolandica*, and some other species of indeterminata microalgae) at a concentration of 80−100 cells/μL. After 10 days of culture at natural temperature (26−32.5°C), four bivalve species, *T*. *gransa* (0.36 ± 0.029 mm^**2**^ in shell width × shell length; mean ± SD, n = 30), *C*. *sinensis* (0.43 ± 0.036 mm^**2**^; mean ± SD, n = 30), *S*. *subcrenata* (0.63 ± 0.037 mm^**2**^; mean ± SD, n = 30) and *S*. *constricta* (0.98 ± 0.041 mm^**2**^; mean ± SD, n = 30), were placed into aquaria containing clean seawater. Bivalves were then starved for 12 h prior to subsequent culture experiment to empty the stomach and avoid the effect of residual diets. Subsequently, same seawater and sea-mud were added into plastic boxes (length 20 × width 15 × depth 6 cm; 1.8 L), and juveniles of about 0.6 g wet weight were sprinkled at a density of 20−25 ind/cm^**2**^. The feeding preferences of four bivalve species and the effects of selected microalgal species on growth of four bivalve species were evaluated in two separate experiments (Fig 1).

#### Feeding preference experiment

In the feeding preference experiment, each treatment was performed in six replicates. A mixture of the four microalgal species (*C*. *calcitrans*, *I*. *galbana*, *P*. *helgolandica* and *N*. *oculata*) in equal proportions was given at the total concentration of 3−4 × 10^**5**^ cells/mL. The suspension was bubbled with filtered air to prevent sedimentation of algae. Experiments lasted for 3 h, and water samples from three repetitions were collected for qPCR analyses at 0-, 0.5-, 1-, 2- and 3-h intervals. To determine the proportion of four microalgal species in biodeposits (pseudofaeces and faeces), larvae from the other three repetitions were removed from the feeding medium after 6 h of feeding. Then biodeposits were collected.

#### Growth experiment

In the growth experiment, unialgal feed was given every day at the concentration of 100−120 cells/μL except for *P*. *helgolandica* (15−20 cells/μL). The suspension was bubbled with filtered air to prevent sedimentation of algae. Experiments lasted for 15 days except for *S*. *constricta* (12 days). The water and mud were renewed every 4 or 5 days, and 10 juveniles were randomly sampled. The shell length and width were determined under the same microscope equipped with a microscale ruler. The shell width was measured as the axis running perpendicular to the hinge line, and the length was measured as the longest line running perpendicular to the axis. Each experimental group was prepared in triplicates. During the feeding trial, the water temperature ranged from 25.2 to 35.5°C.

### Primers and qPCR analysis

#### Microalgal genomic DNA extraction and selection of qPCR primers

To obtain 18S rDNA sequencing of *C*. *calcitrans*, *I*. *galbana*, *P*. *helgolandica* and *N*. *oculata* and determine 18S copy numbers per cell in these microalgal species, 10 mL of the microalgae (the cell number was determined by haemocytometer) were centrifuged at 15,000 rpm for 3 min. Genomic DNA was extracted using a TaKaRa MiniBEST Plant Genomic DNA Extraction Kit (Takara, Japan) following the manufacturer’s instructions.

For 18S rDNA sequencing of *C*. *calcitrans*, *I*. *galbana*, *P*. *helgolandica* and *N*. *oculata*, a universal primer pair (Table 1) was designed based on highly conserved regions from the 18S rDNA sequences of other microalgae available in the GenBank database. Genomic DNA was used as the template for amplification. PCR was performed in a total volume of 25 μl, containing 1 μl of each primer (10 mM), 1 μl of extracted DNA, 2.5 μl of 10X PCR buffer, 4 μl of 1 mM dNTPs, 2.5 mM MgCl_2_, 1 U Taq polymerase (Takara, Japan) and 14 μl of sterilized double-distilled water. The PCR programme was as follows: 95°**C** for 3 min, followed by 35 cycles of 95°**C** for 30 s, 60°**C** for 30 s, and 72°**C** for 1 min. All PCR products were separated on a 1.5% agarose gel and then purified by SanPrep PCR Purification Kit (Sangon Biotech, China). PCR products were cloned into pEASY-T1 simple cloning vector (TransGen, China) and sequenced in Invitrogen (Shanghai, China). Then, species-specific primers targeting to 18S rDNA of the four microalgal species were designed using Primer 5.0 based on the partial 18S rDNA sequences obtained (Table 1).

**Table 1:** Primers used for the cloning of 18S rDNA and qPCR.

Primer set	Forward (5′-3′)	Reverse (5′-3′)	
18S universal primer			
18S	GCTCGNMWYWARGRTTAAGCCATGC	ACCTTGTTASGWCTTCACCYTCCTC	
qPCR primer			
*C*. *calcitrans*	ATGACTTTTCATTGGCGATGGTT		GCCTCTCGGCCAAGGTTTATG
*I*. *galbana*	AACCCCCTGTTGGGGCTC		ACCTCTCGGTCAAGGGAGACG
*P*. *helgolandica*	GTTACTCCTACTTTGGTAGGAGGTGAAC		GGACCTCTCGGTCAAGGTTAGG
*N*. *oculata*	AGTCACGGCCTCTCCGGG		CCTCATGCTTCCATTGGCTAGT

#### qPCR assay

The 18S copy number was quantified using a method described by Durbin et al. [21]. Species-specific primers targeting to 18S rDNA of the four microalgal species were tested with genomic DNA from each species by qPCR to confirm specificity. Plasmids containing 18S rDNA sequences of *C*. *calcitrans*, *I*. *galbana*, *P*. *helgolandica* and *N*. *oculata* were prepared and used as standards for qPCRs. The copy number in standards was calculated from the plasmid concentration (quantified using a NanoDrop ND-2000) and length. Standard curves were generated in a 10-fold serial dilution (1 × 10^**9**^, 1 × 10^**8**^, 1 × 10^**7**^, 1 × 10^**6**^, 1 × 10^**5**^, 1 × 10^**4**^, 1 × 10^**3**^ and 1 × 10^**2**^ copies per reaction). qPCR was carried out in a 25-μL reaction system containing 0.5 μL of each primer (10 mM), 1 μL of genomic DNA template, 12.5 μL of 2× SYBR^**®**^ Premix Ex Taq™ II (Takara, Japan) and 10.5 μL of sterilized double-distilled water on a quantitative thermal cycle (Mastercyclerep *realplex*, Eppendorf, Germany). Briefly, after an initial denaturation step at 95°C for 2 min, the amplification was carried out with 40 cycles at a melting temperature of 95°C for 10 sec, an annealing temperature of 59 ± 2°C for 10 sec, and an extension temperature of 72°C for 20 sec. Melting curve (1.85°C increment/min from 57°C to 95°C) was performed after the amplification phase for confirmation. Each sample was run in triplicate. For each run of unknowns, a duplicate standard curve together and a no template control were conducted. Copy numbers were determined from the Cq value for each microalgal species, template concentration and the standard curve. Amplification efficiency was analyzed according to the equation as follows: E = 10^**(−1 / slope)**^ − 1. To confirm that the PCR efficiency of the plasmid target was the same as the PCR efficiency of the target in the biological sample, the standard curves were also generated with 10-fold serial dilutions of PCR product and extracted DNA. In the present study, the determination of detection limits was based on the method described by Bernardo et al. [22]. We firstly constructed dilution series using plasmids containing target DNA. Each dilution level was conducted with qPCR assays with 20 replicates. The detection limit is defined as lowest dilution where the 20 replicates show 1 failed amplification. For biological sample, the 18S rDNA copy number was normalized using 18S copy numbers per cell, and data were reported as cell number.

### Calculations and statistical analysis

Differences were determined by one way ANOVA using SPSS 16.0 for Windows (SPSS Inc.), and Duncan’s multiple range test was used to inspect differences among four microalgal species. If unequal variance was determined by Levene’s test, data were log-transformed before statistical analysis. Data were expressed as means ± SD, and *P* values of less than 0.05 were considered statistically significant.

## Results

### qPCR assay

#### DNA extraction and design of species-specific qPCR primers

**Fig 2:**
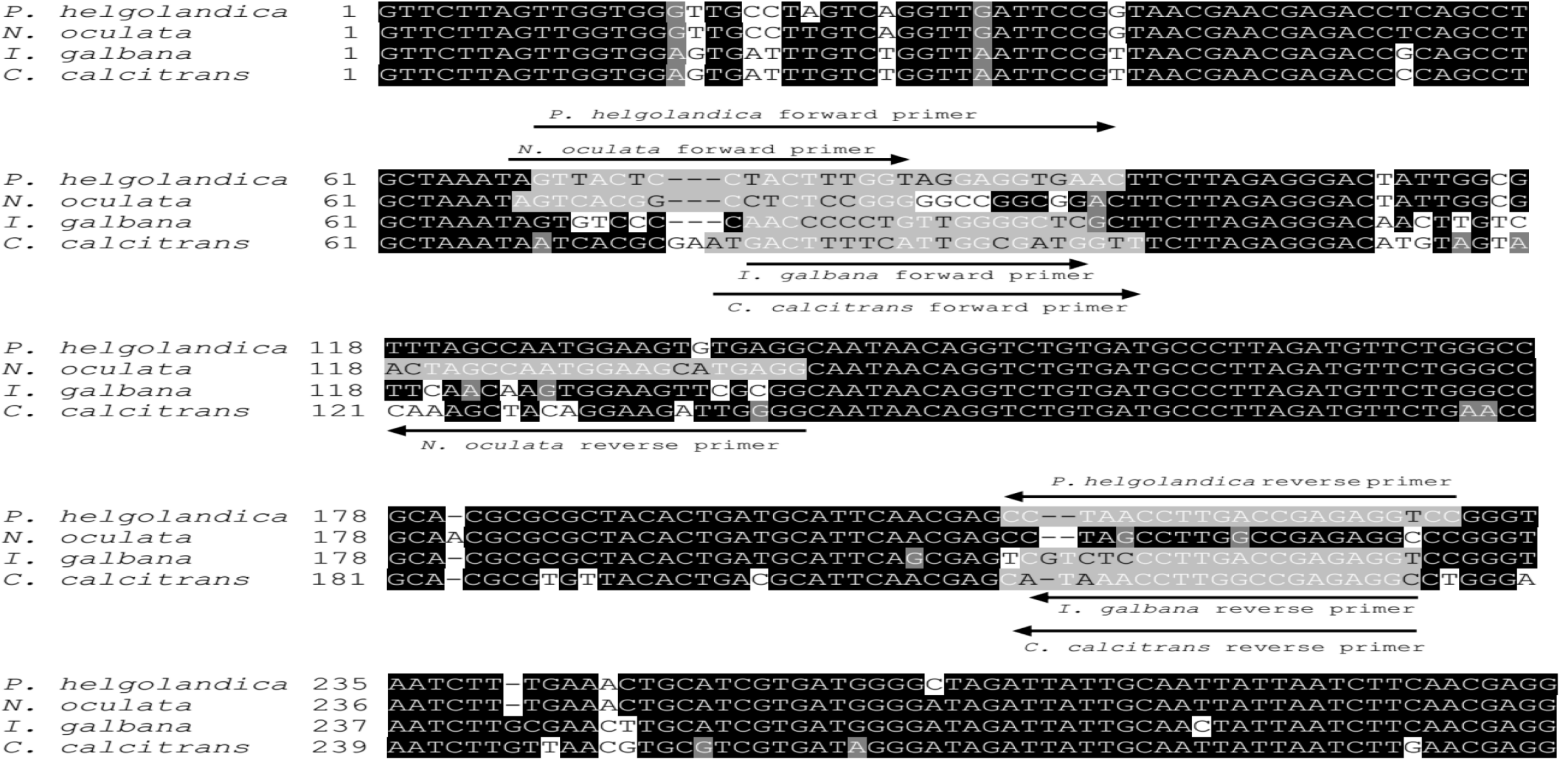
Sequence of 18S rDNA of *Chaetoceros calcitran*, *Isochrysis galbana*, *Platymonas helgolandica* and *Nannochloropsis oculata*. Primers used for qPCR is underlined.

Genomic DNA was extracted from *C*. *calcitrans*, *I*. *galbana*, *P*. *helgolandica* and *N*. *oculata*. About 300 bp of 18S rDNA PCR product was amplified from genomic DNA of *C*. *calcitrans*, *I*. *galbana*, *P*. *helgolandica* and *N*. *oculata* and then sequenced (Fig 2). Species-specific primers targeting to 18S rDNA of the four microalgal species were designed based on the partial 18S rDNA sequences (Fig 2).

#### Assay specificity and sensitivity

**Fig 3:**
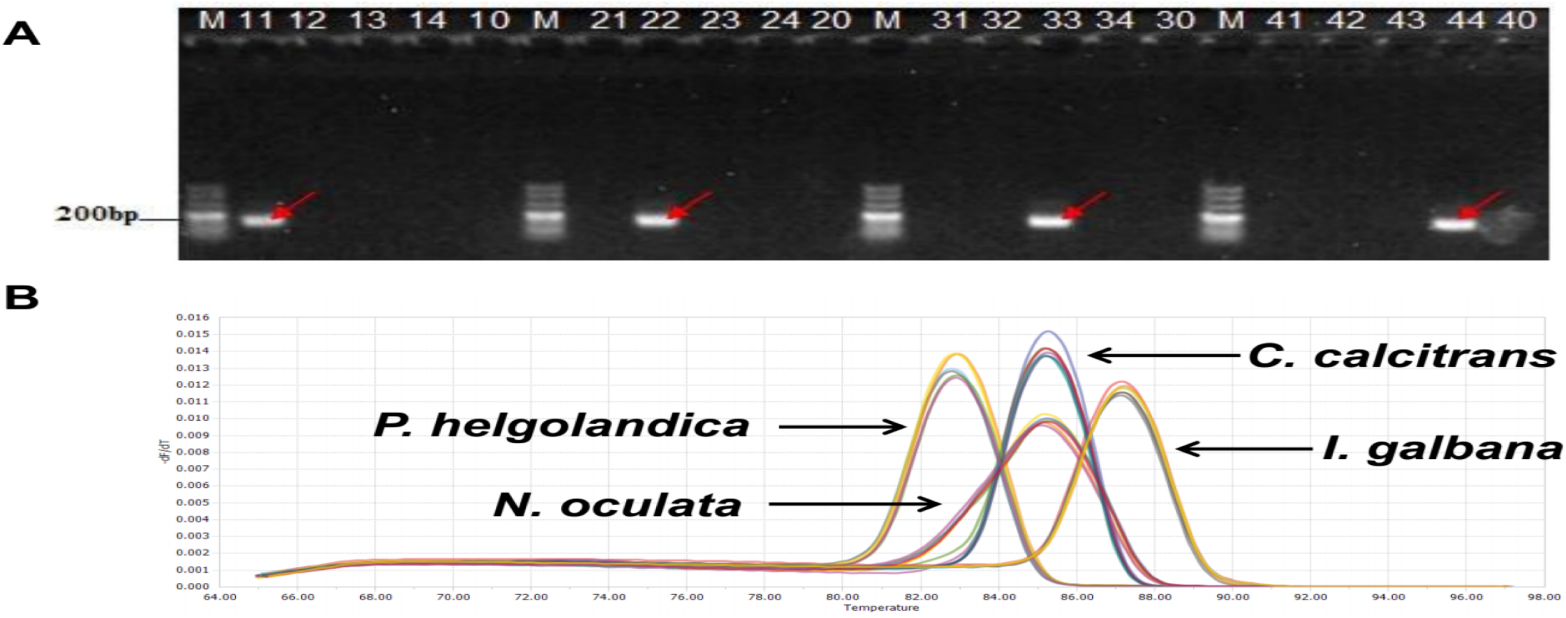
Specificity of the 18S rDNA targeted qPCR primers. (A) PCR products. Lane designations are as follows: M, DNA marker; lanes 10–14, *C*. *calcitrans* primer *+* no DNA, *C*. *calcitrans* DNA, *I*. *galbana* DNA, *P*. *helgolandica* DNA, *N*. *oculata* DNA; lanes 20–24, *I*. *galbana* primer *+* no DNA, *C*. *calcitrans* DNA, *I*. *galbana* DNA, *P*. *helgolandica* DNA, *N*. *oculata* DNA; lanes 30–34, *P*. *helgolandica* primer *+* no DNA, *C*. *calcitrans* DNA, *I*. *galbana* DNA, *P*. *helgolandica* DNA, *N*. *oculata* DNA; lanes 40–44, *N*. *oculata* primer *+* no DNA, *C*. *calcitrans* DNA, *I*. *galbana* DNA, *P*. *helgolandica* DNA, *N*. *oculata* DNA; (B) Melting curve analysis of each qPCR product. The peaks show the melting point of amplified DNA from four microalgal species. The Y-axis is the rate of change of the relative fluorescence units with time (-dF/dT), and the temperature (°C) is indicated on the X-axis.

**Fig 4:**
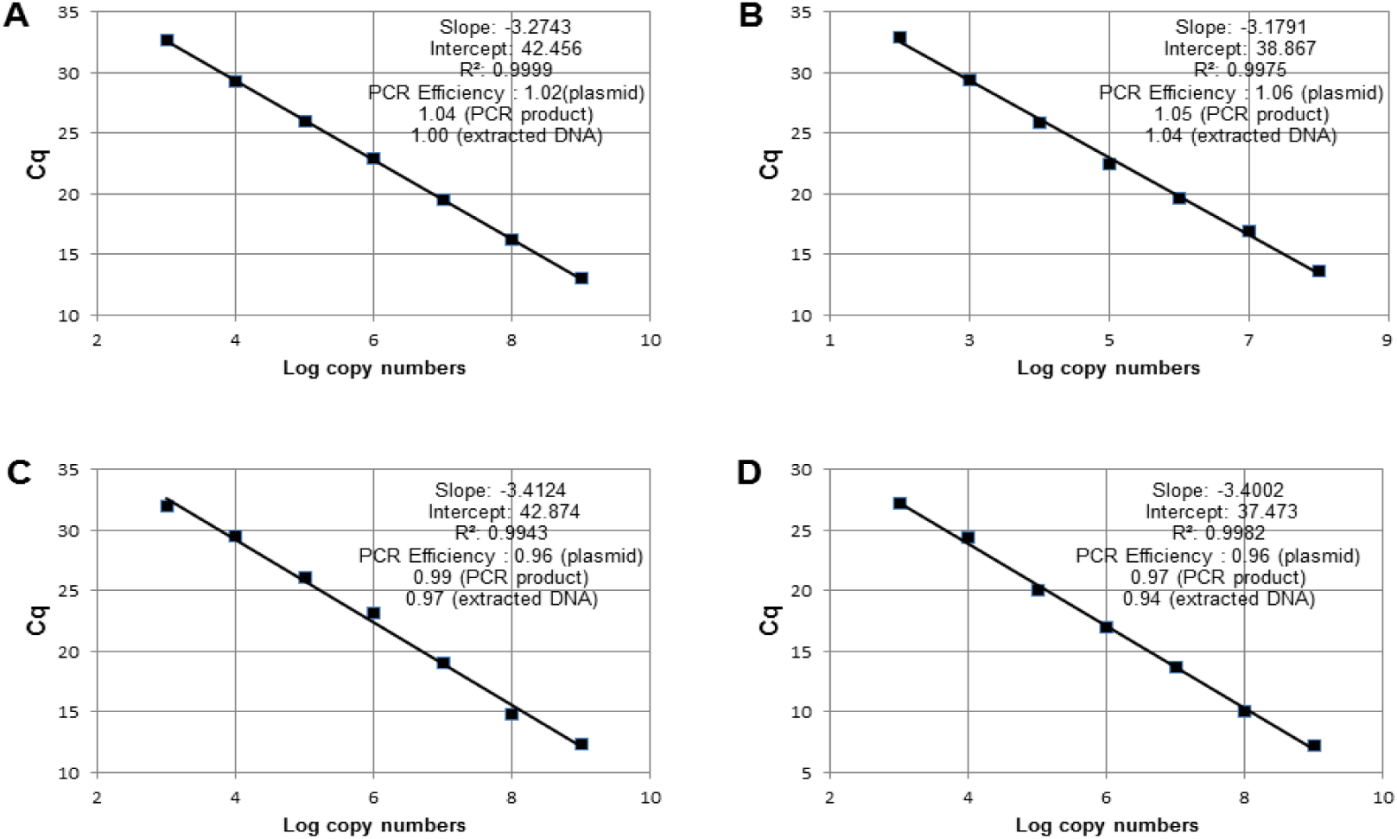
Standard curve of each qPCR primer produced using 10-fold serial dilutions of plasmids containing 18S rDNA sequences of *C*. *calcitrans* (A), *I*. *galbana* (B), *P*. *helgolandica* (C) and *N*. *oculata* (D) as a standard template and values of slope, intercept, correlation (*R*^2^) and PCR efficiencies.

The specificity of the 18S rDNA products was determined by gel electrophoresis (Fig 3A) and melting curve analysis (Fig 3B). A simple regression line of the Cq values for the DNA standards was plotted against the log values of their starting copy numbers. For *C*. *calcitrans*, the amplification efficiency, the intercept value and the r^**2**^ value were 1.02, 42.45 and 0.999, respectively (Fig 4A). For *I*. *galbana*, the amplification efficiency, the intercept value and the r^**2**^ value were 1.06, 38.88 and 0.997, respectively (Fig 4B). For *P*. *helgolandica*, the amplification efficiency, the intercept value and the r^**2**^ value were 0.96, 42.87 and 0.994, respectively (Fig 4C). For *N*. *oculata*, the amplification efficiency, the intercept value and the r^**2**^ value were 0.96, 37.47 and 0.998, respectively (Fig 4D). In this study, the sensitivity of the qPCR was evaluated using 10-fold serially diluted 18S rDNA plasmid containing the target sequence. The minimal detectable amount of 18S rDNA for *C*. *calcitrans*, *I*. *galbana*, *P*. *helgolandica* and *N*. *oculata* was 769, 71, 781 and 21 DNA copies, respectively. The qPCR theoretical detection limit of 3 copies was not reached [23]. We also determined 18S rDNA copy numbers per cell in these four microalgal species under our experimental conditions. A sample of about 1.0×10^**6**^ microalgae (the cell number was determined by haemocytometer) contained approximately 2.99×10^**7**^ copies of *C*. *calcitrans* 18S rDNA, 5.16×10^**6**^ copies of *I*. *galbana* 18S rDNA, 2.05×10^**7**^ copies of *P*. *helgolandica* 18S rDNA and 7.20×10^**6**^ copies of *N*. *oculata* 18S rDNA.

### Sample detection

#### The concentration of each microalgal species in mixed algal suspensions

**Fig 5:**
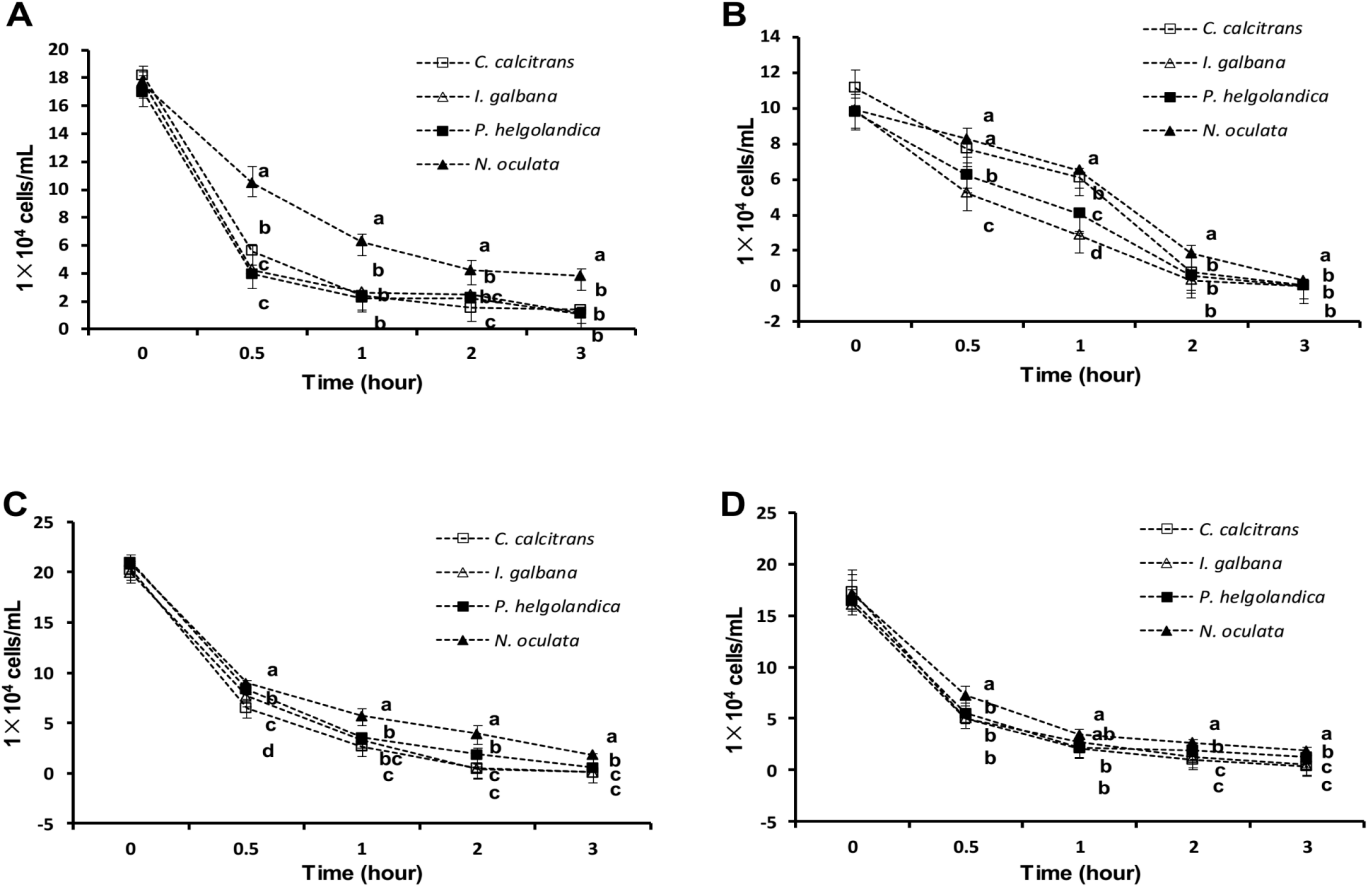
**The concentration of each microalgal species (*Chaetoceros calcitrans*, *Isochrysis galbana*, *Platymonas helgolandica* and *Nannochloropsis oculata*) in the suspensions of *Tegillarca gransa* (A), *Cyclina sinensis* (B), *Scapharca subcrenata* (C) and *Sinonovacula constricta* (D) after 0 h, 0.5 h, 1 h, 2 h and 3 h of feeding.** Values are expressed as the means ± SD (n = 3). Differences between algal species per time point were evaluated by one-way ANOVA, followed by the Duncan’s multiple range test. Labeled means in a column without a common letter differ, *P*<0.05.

Although a mixture of the four microalgal species in equal proportions was given, *N*. *oculata* was higher than *C*. *calcitrans*, *I*. *galbana* and *P*. *helgolandica* in the feeding blends of *T*. *gransa* over the entire duration of the experiment (*P* < 0.05; Fig 5A).

At 0.5 h, the concentration of *C*. *calcitrans* and *N*. *oculata* was highest in the feeding blends of *C*. *sinensis*, followed by *P*. *helgolandica*, whereas the lowest concentration was observed in *I*. *galbana* (*P* < 0.05; Fig 5B). The concentration of *C*. *calcitrans*, *I*. *galbana*, *P*. *helgolandica* and *N*. *oculata* was 6.10±0.36, 2.87±0.12, 4.07±0.15 and 6.50±0.10 (1×10^4^ cells/mL) at 1 h, respectively, and there were significant differences among four groups (*P* < 0.05). The concentration of *N*. *oculata* was higher compared with *C*. *calcitrans*, *I*. *galbana* and *P*. *helgolandica* at 2 h and 3 h (*P* < 0.05).

In the feeding blends of *S*. *subcrenata*, the concentration of *P*. *helgolandica* was lower compared with *N*. *oculata* (*P* < 0.05), whereas it was higher compared with *C*. *calcitrans* and *I*. *galbana* at 0.5 h, 2 h and 3 h (*P* < 0.05; Fig 5C).

In the feeding blends of *S*. *constricta*, the concentration of *N*. *oculata* was higher than *C*. *calcitrans*, *I*. *galbana* and *P*. *helgolandica* at 0.5 h (*P* < 0.05; Fig 5D). At 1 h, the concentration of *N*. *oculata* was higher compared with *C*. *calcitrans* and *P*. *helgolandica* (*P* < 0.05). At 2 h and 3 h, the concentration of *P*. *helgolandica* was lower compared with *N*. *oculata* (*P* < 0.05), whereas it was higher compared with *C*. *calcitrans* and *I*. *galbana* (*P* < 0.05).

#### The concentration of each microalgal species in biodeposits

**Fig 6:**
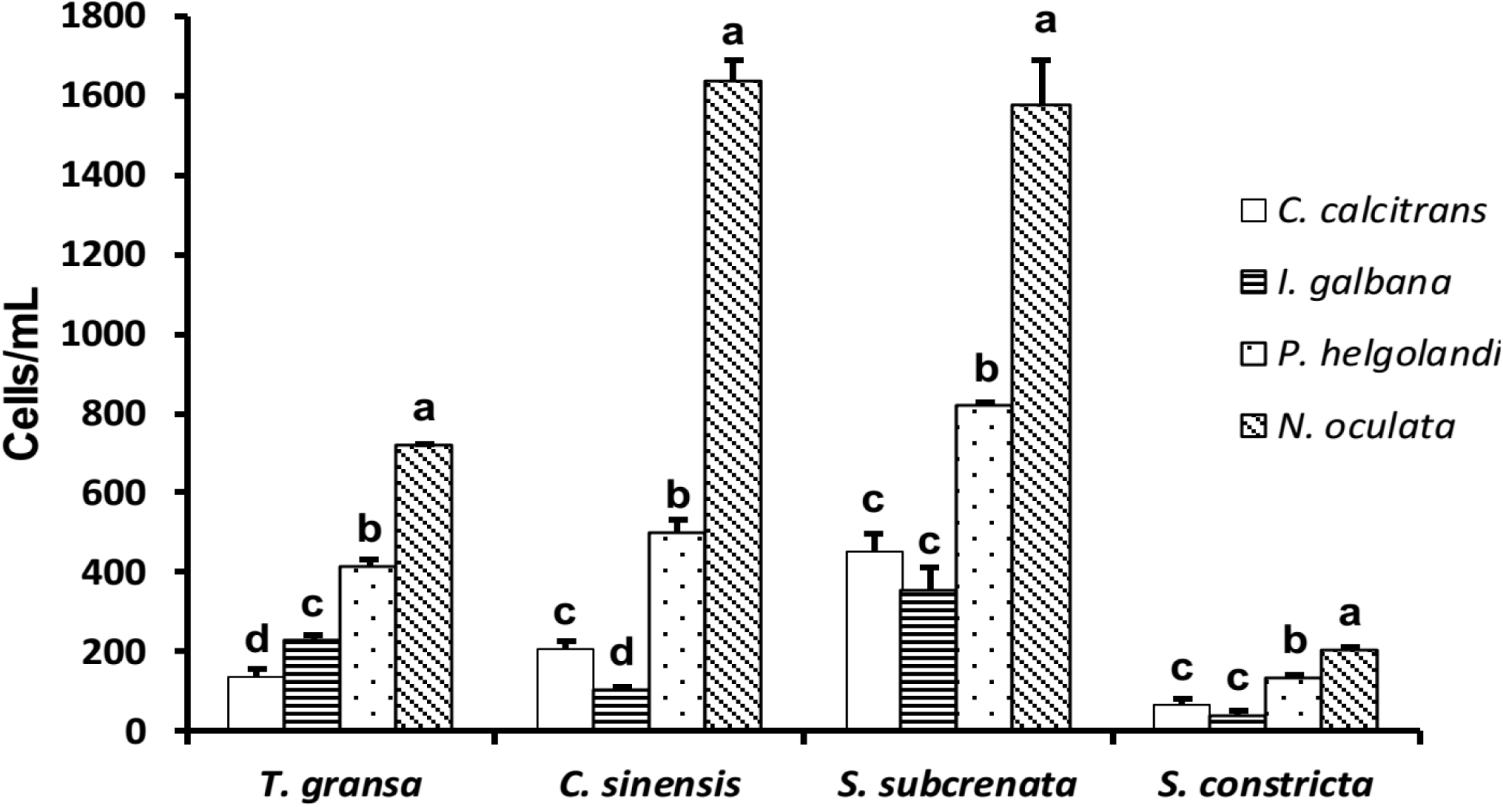
The concentration of each microalgal species (*Chaetoceros calcitrans*, *Isochrysis galbana*, *Platymonas helgolandica* and *Nannochloropsis oculata*) in biodeposits of *Tegillarca gransa*, *Cyclina sinensis*, *Scapharca subcrenata* and *Sinonovacula constricta* after 3 h of feeding. Values are expressed as the means ± SD (n = 3). Differences between algal species per bivalve species were evaluated by one-way ANOVA, followed by the Duncan’s multiple range test. Labeled means without a common letter differ, *P*<0.05.

In biodeposits of *T*. *gransa*, the concentration of *C*. *calcitrans*, *I*. *galbana*, *P*. *helgolandica* and *N*. *oculata* was 136.8±19.9, 228.8±15.8, 414.6±17.4 and 719.7±2.3 cells/mL, respectively, and there were significant differences among four groups (*P* < 0.05; Fig 6). In biodeposits of *C*. *sinensis*, the concentration of *C*. *calcitrans*, *I*. *galbana*, *P*. *helgolandica* and *N*. *oculata* was 208.3±19.8, 106.2±5.7, 501.1±33.3 and 1634.3±52.7 cells/mL, respectively, and there were significant differences among four groups (*P* < 0.05). In biodeposits of *S*. *subcrenata* and *S*. *constricta*, the concentration of *P*. *helgolandica* was lower compared with *N*. *oculata* (*P* < 0.05), whereas it was higher compared with *C*. *calcitrans* and *I*. *galbana* (*P* < 0.05).

### The effects of each microalgal species on growth of four bivalve species

**Fig 7:**
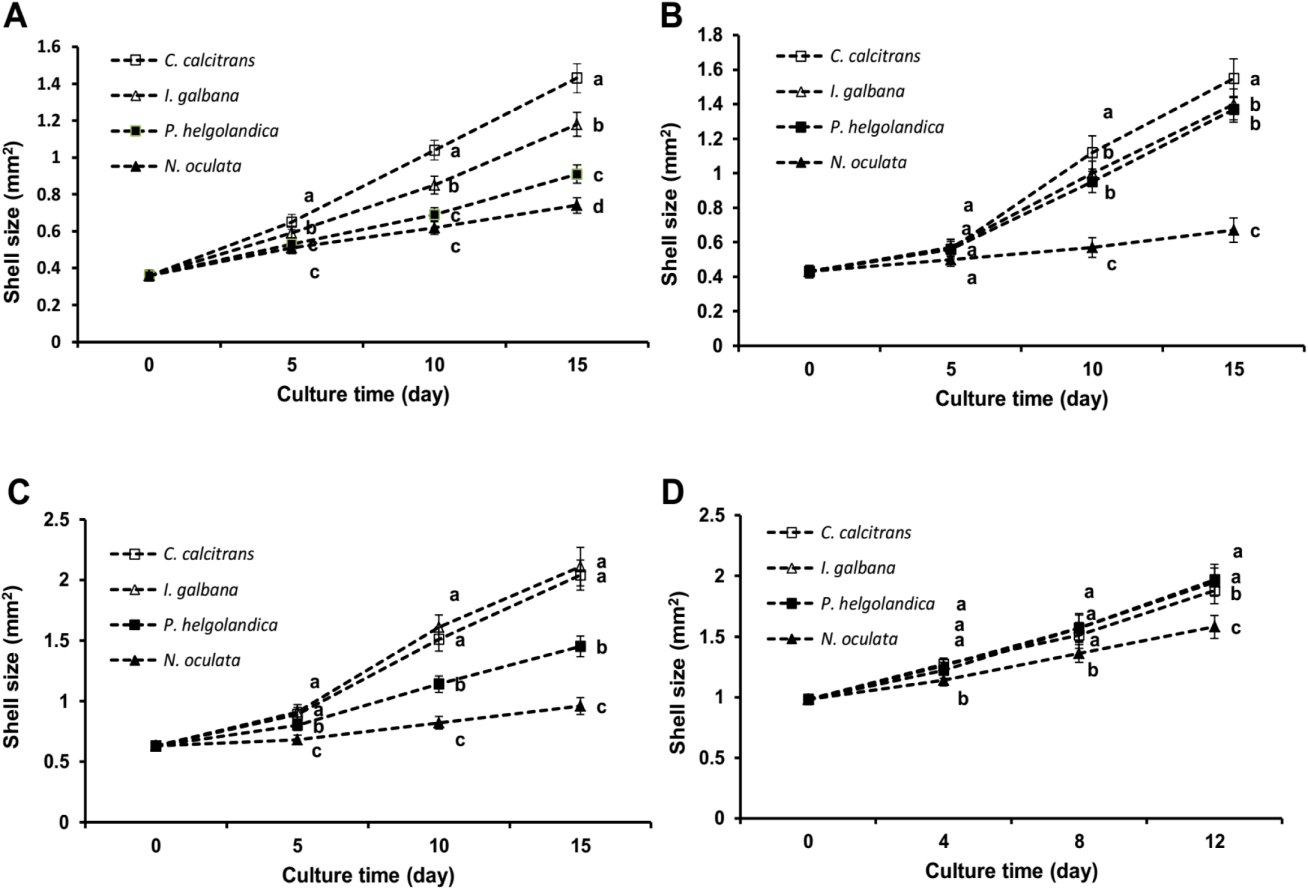
**Growth of *Tegillarca gransa* (A), *Cyclina sinensis* (B), *Scapharca subcrenata* (C) and *Sinonovacula constricta* (D) fed four unialgal diets over 12 days or 15 days.** The shell size is expressed as shell length × shell width (mm^2^). Values are expressed as the means ± SD (n = 3). Statistical significance was evaluated by one-way ANOVA, followed by the Duncan’s multiple range test. Labeled means in a column without a common letter differ, *P*<0.05.

The final shell size of *T*. *gransa* fed *C*. *calcitrans*, *I*. *galbana*, *P*. *helgolandica* and *N*. *oculata* was 1.43±0.078, 1.18±0.065, 0.91±0.050 and 0.74±0.041 mm^2^, respectively, and there were significant differences among four groups (*P* < 0.05; Fig 7A). *C*. *sinensis* fed *C*. *calcitrans* showed the highest growth rate, followed by *I*. *galbana* and *P*. *helgolandica*, whereas the lowest growth rate was observed in *N*. *oculata* (*P* < 0.05; Fig 7B). *S*. *subcrenata* fed *C*. *calcitrans* and *I*. *galbana* showed the highest growth rate, followed by *P*. *helgolandica*, whereas the lowest growth rate was observed in *N*. *oculata* (*P* < 0.05; Fig 7C). *S*. *constricta* fed *I*. *galbana* and *P*. *helgolandica* showed the highest growth rate, followed by *C*. *calcitrans*, whereas the lowest growth rate was observed in *N*. *oculata* (*P* < 0.05; Fig 7D).

## Discussion

Investigation of the selective feeding of bivalves has been challenged by the inaccurate and laborious identification and counting of microalgal species. In the present study, qPCR was used to determine the removal rate of *C*. *calcitrans*, *I*. *galbana*, *P*. *helgolandica* and *N*. *oculata* from mixed algal suspensions of *T*. *gransa*, *C*. *sinensis*, *S*. *subcrenata* and *S*. *constricta*. In addition, we also used this method to quantify the proportion of each microalgal species in biodeposits of four bivalve species. The results indicated that this 18S rDNA-based method was highly specific for each microalgal species. A species-specific primer pair targeting to 18S rDNA exhibited no cross-reaction with other microalgal genomic DNA. Furthermore, the primers designed in our study were sensitive to microalgal genomic DNA extracted from mixed algal suspensions and biodeposits of bivalve larvae. The qPCR enabled the detection of 769 copies of *C*. *calcitrans*18S rDNA, 71 copies of *I*. *galbana* 18S rDNA, 781 copies of *P*. *helgolandica* 18S rDNA and 21 copies of *N*. *oculata*18S rDNA. Our data demonstrated that qPCR was an efficient way to evaluate feeding preferences of bivalve larvae, and such an approach and our findings might be broadly applicable.

Certain bivalves possess the capacity to select particles based on food value [24–26]. In the present study, four bivalve species preferentially removed *C*. *calcitrans* and *I*. *galbana* compared with *N*. *oculata*. Furthermore, the concentration of each microalgal species in biodeposits of all bivalves could be ranked in a descending order as follows: *N*. *oculata* > *P*. *helgolandica* > *C*. *calcitrans* and *I*. *galbana*. These results indicated that *C*. *calcitrans* and *I*. *galbana* were preferentially ingested, whereas *N*. *oculata* was preferentially rejected in biodeposits of all four bivalve species. Accordingly, our growth experiments also suggested that the microalgae preferentially chosen by bivalves resulted in better growth performance. Single-species algal diet of *C*. *calcitrans* or *I*. *galbana* significantly promoted the shell growth of all four bivalve species. Actually, *C*. *calcitrans* and *I*. *galbana* are considered as good feed in many bivalve mollusks, including *Paphies australis* [27], *S*. *constricta* [28] and *T*. *granosa* [29]. In contrast, *N*. *oculata*, commonly used as a diet source in hatcheries of bivalve mollusks, resulted in poorer growth of all four bivalve species. In previous studies, *N*. *oculata* has been also shown to have low food value for *Argopecten nucleus*, *Nodipecten nodosus*, *T*. *granosa* and *S*. *constricta* [17,28–30]. Taken together, these data indicated that qPCR assay might be useful in screening of efficient and reliable microalgal species for each bivalve species. The assay might be effective in improving the bivalve aquaculture and hatchery.

We also attempted to quantify each microalgal species in the stomach and gut contents of four experimental species by qPCR. However, large variations in 18S rDNA copy number were observed from duplicate samples, and experimental data exhibited bad repeatability (data not shown). Previous studies in copepod have indicated that prey DNA was digested very rapidly, and an initial disappearance occurred during the first few minutes after ingestion [14,21]. Therefore, that bivalve larvae in each duplicate was not synchronously sampled and frozen in liquid nitrogen might affect quantitative measurement of 18S rDNA in the stomach and gut contents of bivalves. Future studies need to consider the issue of DNA digestion in the stomach and gut of bivalves, and more research is required to achieve these objectives.

In conclusion, the qPCR utilizing SYBR green developed in this study was a feasible technique for detection of the selective feeding of bivalve larvae. The qPCR assay revealed good specificity and sensitivity. Using the assay, we found that *C*. *calcitrans* and *I*. *galbana* could significantly promote shell growth as preferentially ingested diets, whereas *N*. *oculata*, as preferentially rejected diet, resulted in poorer growth in four bivalve species.
